# Letter to the Editor: The potential role of intraoperative flow cytometry during recurrent glioblastoma surgery

**DOI:** 10.1016/j.bas.2026.106273

**Published:** 2026-08-07

**Authors:** Eleni Romeo, George Vartholomatos, George Markopoulos, Spyridon Voulgaris, George A. Alexiou

**Affiliations:** a Department of Neurosurgery, University Hospital of Ioannina, Ioannina, Greece; b Neurosurgical Institute, University of Ioannina, Ioannina, Greece; c Haematology Laboratory, Unit of Molecular Biology and Translational Flow Cytometry, Ioannina, 45110, Greece

To the editor:

We read with great interest the retrospective study by Alhalabi et al. evaluating the utility of intraoperative magnetic resonance imaging (iMRI) and volumetric analysis in surgery for recurrent glioblastoma (GBM). The study included 150 patients with recurrent GBM who underwent iMRI-guided re-resection. The authors demonstrated that iMRI prompted additional resection in 77% of cases and increased the proportion of RANO class 1–2 resections from 58% on intraoperative imaging to 85% on early postoperative MRI. Importantly, additional resection was not associated with an increased rate of either transient or permanent neurological deficits. Of particular interest, gross total resection (GTR), defined as a residual tumor volume of ≤0.175 mL, was independently associated with improved survival following re-resection (Alhalabi et al., 2026).

However, one of the greatest challenges in recurrent GBM surgery is the intraoperative differentiation of recurrent tumor from radiation necrosis and other treatment-related changes (Patel and Chavda, 2023). This issue is particularly relevant to the present study, as only patients with histopathologically confirmed recurrent tumor were included, whereas cases with radiation-induced necrosis, either alone or in combination with viable tumor, were excluded from the analysis (Alhalabi et al., 2026). Thus, although iMRI provides accurate anatomical information it may not always determine the biological nature of that tissue.

In this context, we believe that intraoperative flow cytometry (iFC) may serve as a valuable adjunct for the intraoperative evaluation of recurrent GBM. Unlike imaging techniques, iFC provides direct biological information by rapidly assessing DNA index and cell-cycle distribution from a small tissue specimen (Alexiou et al., 2015). Using the Ioannina protocol, our group has demonstrated that iFC can differentiate tumor core from peritumoral tissue within minutes and can contribute to the intraoperative assessment of tumor grade, tumor type, and surgical margins (Alexiou et al., 2015; Vartholomatos et al., 2023; Romeo et al., 2026).

The biological significance of iFC is further supported by our previous studies using 99mTc-tetrofosmin SPECT. We demonstrated a significant positive correlation between (99m)Tc-tetrofosmin uptake and the percentage of tumor cells in the S phase of the cell cycle, as determined by flow cytometry (Alexiou et al., 2009). Furthermore, 99mTc-tetrofosmin SPECT showed potential in differentiating recurrent glioma from radiation necrosis (Alexiou et al., 2007). These findings suggest that flow cytometric proliferative indices reflect the biological activity of gliomas (Alexiou et al., 2009). Based on this association, we can hypothesize that iFCmay also provide useful information for distinguishing viable recurrent tumor from radiation-induced changes during recurrent GBM surgery.

In addition, while iMRI provides precise anatomical information regarding residual contrast-enhancing tissue, iFC may offer immediate biological characterization of the sampled tissue, potentially improving intraoperative decision-making during re-resection. Unlike iMRI, iFC is rapid, inexpensive, and does not prolong the surgical procedure or require major modifications to the operative workflow (Romeo et al., 2025). Moreover, iFC may represent a widely accessible technique for neurosurgical centers, as flow cytometers are already available in many hospitals and research institutions (Romeo et al., 2025).

For these reasons, prospective multicenter studies are needed to validate the clinical utility of iFC in recurrent GBM surgery, particularly for intraoperative margin assessment and the differentiation of viable recurrent tumor from treatment-related changes.

## Conflict of interest

None.
